# New species of *Synodontella* (Monogenea, Ancyrocephalidae) gill parasites of two *Synodontis* spp. (Pisces, Mochokidae) from the Boumba River (Congo Basin, East Cameroon)

**DOI:** 10.1051/parasite/2019037

**Published:** 2019-06-27

**Authors:** Jonathan A. Mbondo, Jacques Nack, Arnold R. Bitja Nyom, Antoine Pariselle, Charles F. Bilong Bilong

**Affiliations:** 1 University of Yaoundé 1, Laboratory of Parasitology and Ecology PO Box 812 Yaoundé Cameroon; 2 Specialized Research Center for Marine Ecosystems, IRAD PO Box 219 Kribi Cameroon; 3 University of Douala, Laboratory of Parasitology and Ecology PO Box 24157 Douala Cameroon; 4 Institute of Fisheries and Aquatic Sciences of Yabassi (ISH), University of Douala Cameroon; 5 Department of Biological Sciences, the University of Ngaoundéré PO Box 454 Ngaoundéré Cameroon; 6 ISEM, Univ. Montpellier, CNRS, IRD Montpellier France; 7 Faculty of Sciences, Laboratory “Biodiversity, Ecology and Genome”, Research Center “Plant and Microbial Biotechnology, Biodiversity and Environment”, Mohammed V University in Rabat Morocco

**Keywords:** *Synodontella angustupenis* n. sp., *Synodontella simplex* n. sp., *Synodontella longipenis* n. sp., *Synodontis*, Siluriformes, Africa

## Abstract

Three new species of *Synodontella* Dossou & Euzet, 1993 are described from two species of *Synodontis* (Mochokidae) collected from the middle course of the Boumba River (East Cameroon): *Synodontella angustupenis* n. sp. from *Synodontis nummifer*, *Synodontella longipenis* n. sp. and *Synodontella simplex* n. sp. from *Synodontis decorus*. These new species are different from the other *Synodontella* species already described due to their horseshoe-shaped dorsal transverse bars. *Synodontella angustupenis* differs from *S. longipenis* and *S. simplex* by the morphology of its penis, a thin tube, and its accessory piece, bifid at its extremity. *Synodontella longipenis* differs from the other two species by the morphology and the size of its male copulatory organ, which is very long. *Synodontella simplex* differs from *S. angustupenis* and *S. longipenis* by the shape of its penis, which is simple, and of its accessory piece, with a developed heel. The difference between the dorsal transverse bars of *Synodontella* species from the Sanaga River in Cameroon (and other localities in Africa), slightly curved, and those from the Boumba River, horseshoe-shaped, makes it possible to separate *Synodontella* species into two different subgroups. This difference can be explained by a long isolation period of the hosts, living in different river basins, followed by the divergence of the parasite populations (vicariant speciation).

## Abstract (fr)

Trois nouvelles espèces de *Synodontella* Dossou & Euzet, 1993 sont décrites chez deux espèces de *Synodontis* (Mochokidae) collectées dans le cours moyen de la rivière Boumba (Est Cameroun) : *Synodontella angustupenis* n. sp. de *Synodontis nummifer*, *Synodontella longipenis* n. sp. et *Synodontella simplex* n. sp. de *Synodontis decorus*. Ces nouvelles espèces sont différentes des autres espèces de *Synodontella* déjà décrites par leurs barres transversales dorsales en forme de fer à cheval. *Synodontella angustupenis* diffère de *S. longipenis* et de *S. simplex* par la morphologie de son pénis, un tube fin et de sa pièce accessoire, bifide à son extrémité. *Synodontella longipenis* diffère des deux autres espèces par la morphologie et la taille de son organe copulateur mâle, qui est très long. *Synodontella simplex* se distingue de *S. angustupenis* et *S. longipenis* par la forme de son pénis, qui est simple, et de sa pièce accessoire, avec un talon développé. La différence entre les barres transversales dorsales des espèces de *Synodontella* du fleuve Sanaga au Cameroun (et d’autres localités africaines), légèrement incurvées, et celles de la rivière Boumba, en forme de fer à cheval, permet de séparer les espèces de *Synodontella* en deux sous-groupes différents. Cette différence peut être expliquée par une longue période d’isolement des hôtes, vivant dans différents bassins hydrographiques, suivie de la divergence des populations de parasites (spéciation vicariante).

## Introduction

The high incidence of fish diseases remains a major constraint for the successful economic development of cultured species, and ectoparasites such as monogeneans are probably the cause of such pathologies [16]. Members of the catfish family Mochokidae are amongst the most important teleost species suitable for aquaculture, and species of *Synodontis* Cuvier, 1817 are of great commercial importance in Africa [7]. Taking advantage of a sampling campaign for the study of the Congo basin’s fish species in the East Region of Cameroon, we examined *Synodontis* species; among the 51 valid species reported, 48 are endemic [3], but their culture potential or parasitic fauna are not well known. Gill filaments of the two local sampled species were studied for their specific monogenean parasites belonging to *Synodontella* Dossou & Euzet, 1993 [4]. The type species of this genus, *Synodontella synodontii* (Paperna & Thurston, 1968), had been assigned and later reassigned to different ancyrocephalid genera, *Ancyrocephalus* Creplin, 1839, then *Schilbetrema* Paperna & Thurston, 1968 [4]. However, this species presents unusual morphological characteristics by having a ventral anchor with a hull and a welded central protuberance of the ventral bar; thus, Dossou and Euzet [4] proposed the genus *Synodontella* to include monogeneans with these features. The morphology of the haptoral sclerites of *Synodontella* spp. is close to that of two other ancyrocephalid genera: *Schilbetrema* by the shape of the ventral and dorsal anchors and by having a central protuberance on the ventral bar; *Protoancylodiscoides* by the morphology of the ventral anchors with a hull. The first molecular data on *Synodontella* have shown that this taxon is closer to *Schilbetrema* than to the other dactylogyrid genera parasitizing catfishes, namely *Quadriacanthus* Paperna, 1961, *Thaparocleidus* Jain, 1952 or *Pseudancylodiscoides* Yamaguti, 1963 [13]. To date, only seven species of *Synodontella* have been reported, all from Africa, i.e.: *Synodontella synodontii* [12] described from the gills of *Synodontis victoriae* Boulenger, 1906; *Synodontella arcopenis* Dossou & Euzet, 1993 [4] from *Synodontis sorex* Günther, 1864; *Synodontella melanoptera* Dossou & Euzet, 1993 [4] from *Synodontis melanopterus* Boulenger, 1902; *Synodontella davidi* Dossou & Euzet, 1993 [4] from *Synodontis membranaceus* (Geoffroy St Hilaire, 1809); *Synodontella zambezensis* Douëllou & Chishawa, 1995 [5] from *Synodontis zambezensis* Peters, 1852; *Synodontella apertipenis* Mbondo, Nack & Pariselle, 2017 [11] and *Synodontella sanagaensis* Mbondo, Nack & Pariselle, 2017 [11] from *Synodontis rebeli* Holly, 1926. The present parasitological survey on *Synodontis* spp. provides three new species of *Synodontella* described herein*.* The diversification of Cameroonian *Synodontella* species is also discussed.

## Materials and methods

**Figure 1:**
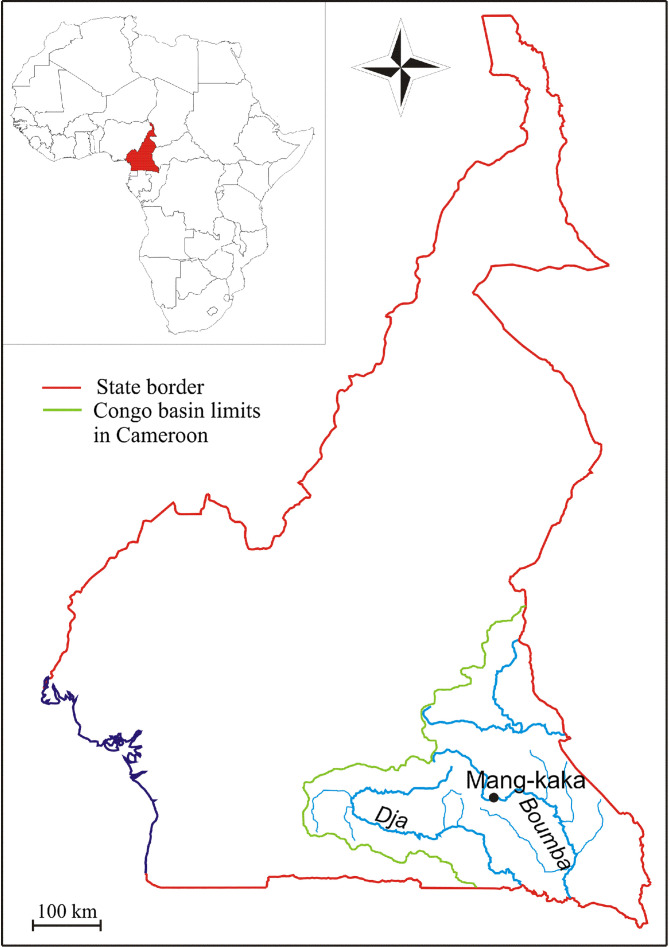
Map of the Boumba River with sampling locality.

Fish specimens (5 *Synodontis nummifer* Boulenger, 1899, 132–174 mm SL; and 9 *S. decorus* Boulenger, 1899, 125–246 mm SL) were caught (02/2017) by angling from the middle course of the Boumba River near Mang-kaka [3° 18′42.89″N 14° 04′43.19″E (see Fig. 1)]. Fishes, euthanized by severing their dorsal spine, were dissected on site; gill arches were removed by dorsal and ventral sections, and then placed in a Petri-dish containing tap water. Monogeneans were dislodged from the gill filaments with the aid of a dissecting needle and were mounted between slide and cover slip in a drop of glycerin ammonium-picrate (GAP) mixture [10]. Preparations were sealed using Glyceel [1] and returned to the Laboratory of Parasitology and Ecology of the University of Yaoundé I for further laboratory analyses. Morphological description and measurements of the sclerotized pieces of the haptor and the male copulatory organ (MCO) were carried out according to Dossou & Euzet (1993) using a Leica DM2500 microscope and LAS 3.8 software. Drawings of the sclerotized pieces were carried out using Coral Draw X4 Software (Ver 14.0.0.701; Corel Corporation, www.corel.com). Type specimens were deposited at the Royal Museum for Central Africa (RMCA, Tervuren) and at the Muséum National d’Histoire Naturelle (MNHN, Paris).

## Results

The general appearance of all the species described corresponds to the diagnosis of *Synodontella* given by Dossou & Euzet (1993).

### *Synodontella angustupenis* Mbondo, Nack & Pariselle n. sp.

urn:lsid:zoobank.org:act:29D5F9CE-8907-4A65-9E0A-3D531BF065F7

*Type host*: *Synodontis nummifer* Boulenger, 1899.

*Site*: Gills.

*Type-locality*: Mang-kaka, Cameroon (3° 18′42.89″N 14° 04′43.19″E).

*Prevalence*: 80%.

*Mean intensity*: 4.6.

*Material*: Twelve whole-mounted specimens in GAP solution.

*Type specimens*: Holotype: RMCA No. M.T. 38597; Paratypes: RMCA No. M.T.38598-38599, MNHN HEL1020-HEL1021.

*Etymology*: The specific epithet (an adjective) is from Latin (*Angustus* = small + *penis* = penis) and refers to the small size of the MCO of the members of this species.

*Note*: The authors of the new taxon are different from the authors of this paper; Article 50.1 and Recommendation 50A of International Code of Zoological Nomenclature [9].

**Figure 2:**
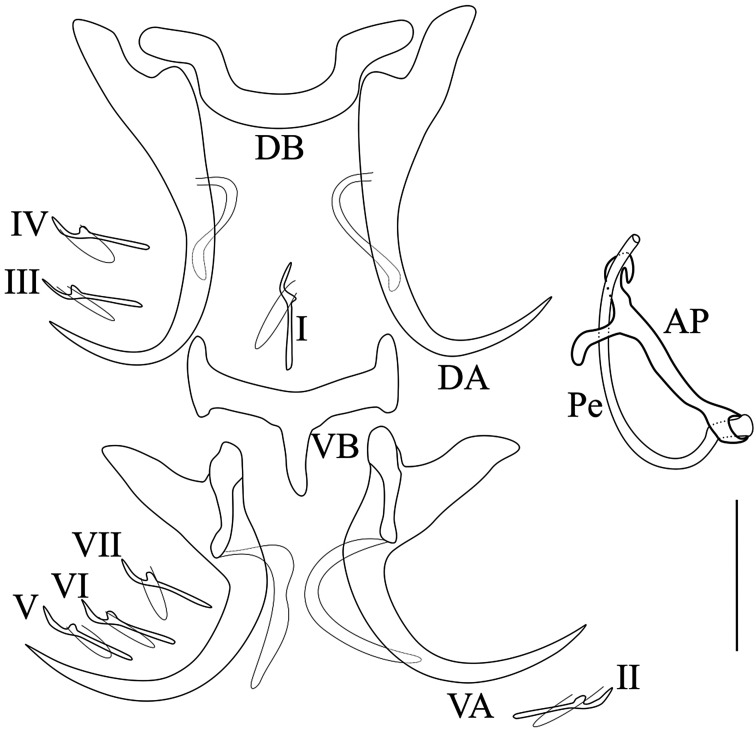
Sclerotized parts of *Synodontella angustupenis* n. sp. Scale bar = 20 μm. AP, accessory piece; DA, dorsal anchor; DB, dorsal bar; VA, ventral anchor; VB, ventral bar; Pe, penis; I-VII, hooks.

*Description* (Table 1, Fig. 2): Body flattened dorso-ventrally; length 485.7–743; width 108.1–147.2. Two pairs of eye-spots of equal size, anterior to pharynx. Haptor with two pairs of anchors; dorsal ones larger with base sabot-shaped and reduced shaft. Dorsal bar horseshoe-shaped. Ventral anchors with a developed guard; diagonal hull with thin sclerotized filament at posterior end. Ventral transverse bar with central posterior protuberance; two lateral arms with enlarged ends. Seven pairs of small hooks, approximatively equal in size, and retained larval appearance. The male copulatory organ bow shaped; thin tubular penis; accessory piece straight, welded to the base of the penis and bifid at the extremity.

**Table 1:** Measurements of the three new species (in µm: mean (minimum-maximum)).

Specific names	*Synodontella angustupenis* n. sp.	*Synodontella longipenis* n. sp.	*Synodontella simplex* n. sp.
Number of specimens	12	07	22
Total length	618.2 (485.7–743)	1024.6 (508.7–1304.9)	801.9 (591.3–1146.7)
Width	123 (108.1–147.2)	164.8 (146.3–194.7)	123.4 (92–163.3)
Dorsal anchor			
a	48.4 (45.2–50.4)	64.9 (63.6–66.5)	56.2 (54.1–58.7)
b	38.4 (37.4–40)	54.6 (52–56.2)	46.2 (43.2–48.5)
c	3.6 (3.2–4.1)	5.1 (4.3–6.1)	3.9 (3–4.5)
d	15.5 (15–16.3)	17.6 (16.9–18.4)	17.1 (14.9–18.4)
e	16.8 (16.2–18.6)	18.9 (18.1–20)	18.3 (17–19.8)
Dorsal bar			
x	35.3 (33.8–36.6)	47.4 (45.5–48.9)	53.3 (50–55.8)
w	4.8 (4.4–5.2)	8.4 (7.8–8.7)	7.8 (6.8–9.3)
Ventral anchor			
a	25.1 (23.4–26.9)	46.9 (45.2–47.6)	42.8 (40.7–44.4)
b	28 (26.2–29.7)	39.3 (38.6–39.7)	35.6 (32.4–38.3)
c	6.2 (5.5–6.9)	8.1 (7.3–8.7)	8.5 (7.6–9.4)
d	14.7 (14.2–15.6)	16.3 (14.4–17.4)	14.3 (12.3–16.1)
e	22.7 (21.4–24.2)	29.2 (28–29.7)	25.9 (23.1–27.9)
Ventral bar			
x	29.9 (28.4–32.6)	47.3 (46.4–48.1)	54.4 (51.2–57.5)
w	5.4 (4.2–6.3)	7 (6.5–7.5)	8.8 (6–10.1)
h	8.7 (8–9.3)	11.7 (10.2–13)	11.9 (10–13.5)
Male copulatory organ			
Pe	45.8 (42.2–57.5)	162.9 (160.6–165.7)	49.7 (47.2–52.9)
Ap	24.1 (22.2–27.5)	85 (82.3–86.6)	65.4 (62–69.3)

*Remarks*: *Synodontella angustupenis* n. sp. differs from all other known *Synodontella* spp. by the shape of the dorsal transverse bar (horseshoe-shaped) and of its accessory piece (straight and bifid at its distal extremity).

### *Synodontella longipenis* Mbondo, Nack & Pariselle n. sp.

urn:lsid:zoobank.org:act:998B238D-CEDC-4590-906B-DBE743D4DE3B

*Type host*: *Synodontis decorus* Boulenger, 1899.

*Site*: Gills.

*Type-locality*: Mang-kaka, Cameroon (3° 18′42.89″N 14° 04′43.19″E).

*Prevalence*: 22.2%.

*Mean intensity*: 0.55.

*Material*: Seven whole-mounted specimens in GAP solution.

*Type specimens*: Holotype: RMCA No. M.T. 38592; Paratypes: RMCA No. M.T. 38593-98594, MNHN HEL1022-HEL1023.

*Etymology*: The specific name is from Latin (*longi(s)* = long + *penis* = penis) and refers to the great length of the MCO.

*Note*: The authors of the new taxon are different from the authors of this paper; Article 50.1 and Recommendation 50A of International Code of Zoological Nomenclature [9].

**Figure 3:**
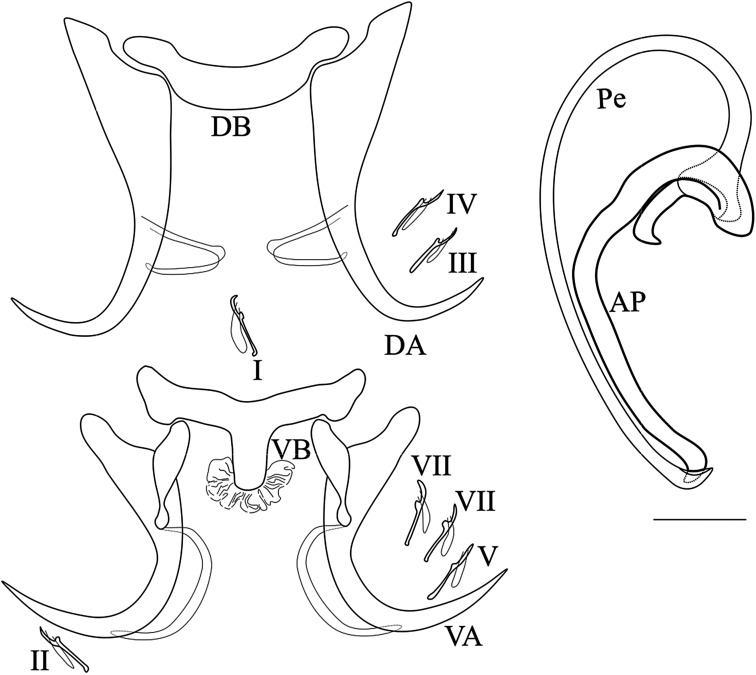
Sclerotized parts of *Synodontella longipenis* n. sp. Scale bar = 20 μm. AP, accessory piece; DA, dorsal anchor; DB, dorsal bar; VA, ventral anchor; VB, ventral bar; Pe, penis; I-VII, hooks.

*Description* (Table 1, Fig. 3): Body flattened dorso-ventrally; length 508.7–1304.9; width 146.3–194.7. Two pairs of eye-spots, anterior to pharynx, of equal size. Haptor separated from the rest of the body by a narrow constriction and made up of two pairs of anchors. Dorsal anchors with long blade, bent at third; shaft rudimentary; thin sclerotized filaments present. Dorsal bar horseshoe-shaped. Ventral anchors smaller than dorsal ones; hull with a thin sclerotized filament at posterior end. Ventral bar with central protuberance crowned by a cuticular structure and enlarged ends. 14 small hooks, approximatively equal in size and retained larval appearance. The male copulatory organ consisting of a long copulatory tube (penis), curved at proximal part and folded at 90° at distal extremity; and a well sclerotized bifid accessory piece, with one long and one short arm, folded around the basal extremity of the penis, both ending hook-shaped.

*Remarks*: *Synodontella longipenis* is close to *Synodontella davidi* by the morphology of the MCO, in both species the penis is a long curved tube; the accessory piece is also long and welded around the penis base. *Synodontella longipenis* differs from *S. davidi* by the size of the penis (163 *vs*. 120) and by having a dorsal bar horseshoe-shaped and the central protuberance of the ventral bar crowned by a cuticular structure. *Synodontella longipenis* resembles *S. angustupenis* by having a dorsal bar horseshoe-shaped, but differs by having a much longer penis and accessory piece (163 *vs*. 46 and 85 *vs.* 24, respectively) and a ventral bar central protuberance crowned by a cuticular structure.

### *Synodontella simplex* Mbondo, Nack & Pariselle n. sp.

urn:lsid:zoobank.org:act:DBB06616-9935-4447-8602-FEBB1ABCBFB3

*Type host*: *Synodontis decorus* Boulenger, 1899.

*Site*: Gills

*Type-locality*: Mang-kaka, Cameroon (3° 18′42.89″N 14° 04′43.19″E)

*Prevalence*: 66.6%

*Mean intensity*: 2.4

*Material*: twenty-two whole-mounted specimens in GAP solution.

*Type specimens*: Holotype: RMCA No. M.T.38596, Paratypes: RMCA No. M.T. 38595, MNHN HEL1022-HEL1023.

*Etymology*: The specific name is from Latin (*simplex* = simple) and refers to the simple shape of the MCO.

*Note*: The authors of the new taxon are different from the authors of this paper; Article 50.1 and Recommendation 50A of International Code of Zoological Nomenclature [9].

**Figure 4:**
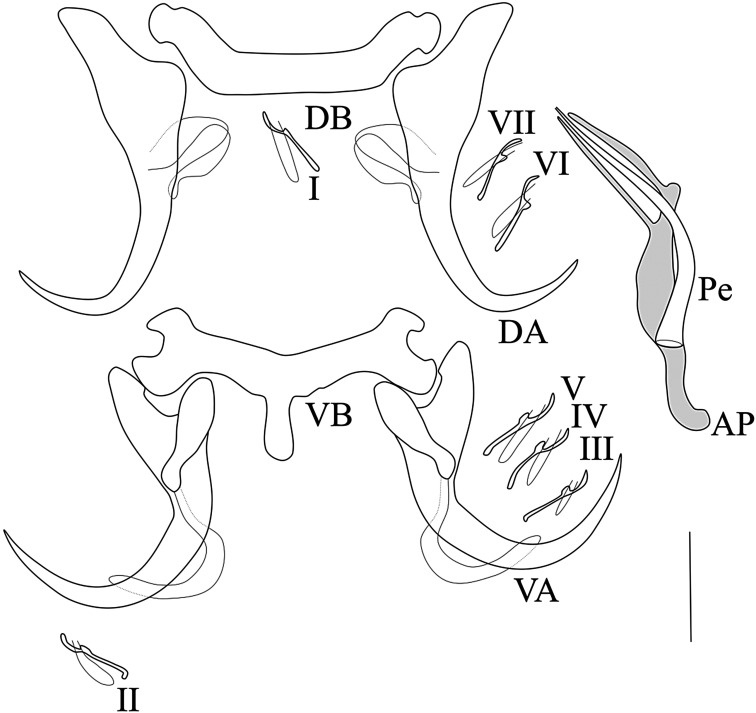
Sclerotized parts of *Synodontella simplex* n. sp. Scale bar = 20 μm. AP, accessory piece; DA, dorsal anchor; DB, dorsal bar; VA, ventral anchor; VB, ventral bar; Pe, penis; I-VII, hooks.

*Description* (Table 1, Fig. 4): Body flattened dorso-ventrally; length 591.3–1146.7; width 92–163.3; two pairs of eye-spots, anterior to the pharynx and of equal size. Haptor separate of the rest of the body by a narrow constriction and made up of two pairs of anchors. Dorsal anchors with base enlarged, small and slightly sclerotized filament present; reduced shaft. Dorsal bar horseshoe-shaped. Ventral anchors smaller than dorsal ones, developed guard, hull with slightly sclerotized filament at distal end. Ventral bar with central protuberance and lateral arms with spanner-shaped ends. Fourteen (14) small hooks, approximatively equal in size and retain larval appearance. The male copulatory organ made up of a simple penis with slightly flared base and narrowed at distal end; well sclerotized accessory piece, longer than the penis and bifurcated at distal extremity; proximal heel present and well developed.

*Remarks*: *Synodontella simplex* n. sp. differs from all other known *Synodontella* spp. by the morphology of the MCO made up of a simple penis, slightly flared at the base (with no visible bulb), and a well sclerotized accessory piece, longer than the penis, with a bifid distal end and a developed heel at the proximal extremity; this MCO morphology had never been observed before in *Synodontella* spp.

## Discussion

**Figure 5:**
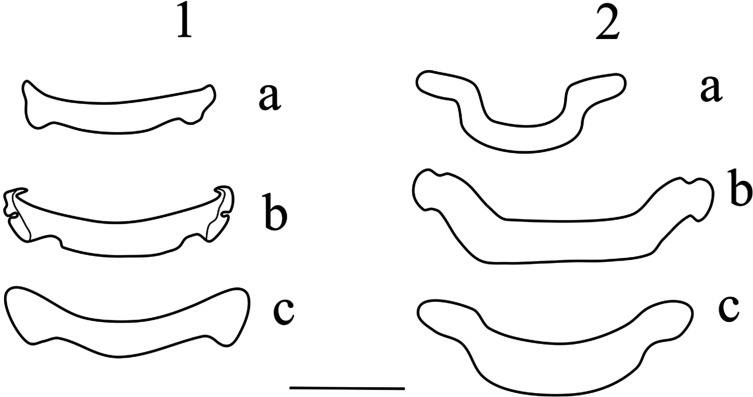
Comparison of the dorsal bar shapes of *Synodontella* species from the Sanaga River (1) and from the Boumba River (2). 1.a, *Synodontella melanoptera*; 1.b, *Synodontella apertipenis*; 1.c, *Synodontella sanagaensis*; 2.a, *Synodontella angustupenis* n. sp.; 2.b, *Synodontella simplex* n. sp.; 2.c, *Synodontella longipenis* n. sp. Scale bar = 20 μm.

**Figure 6:**
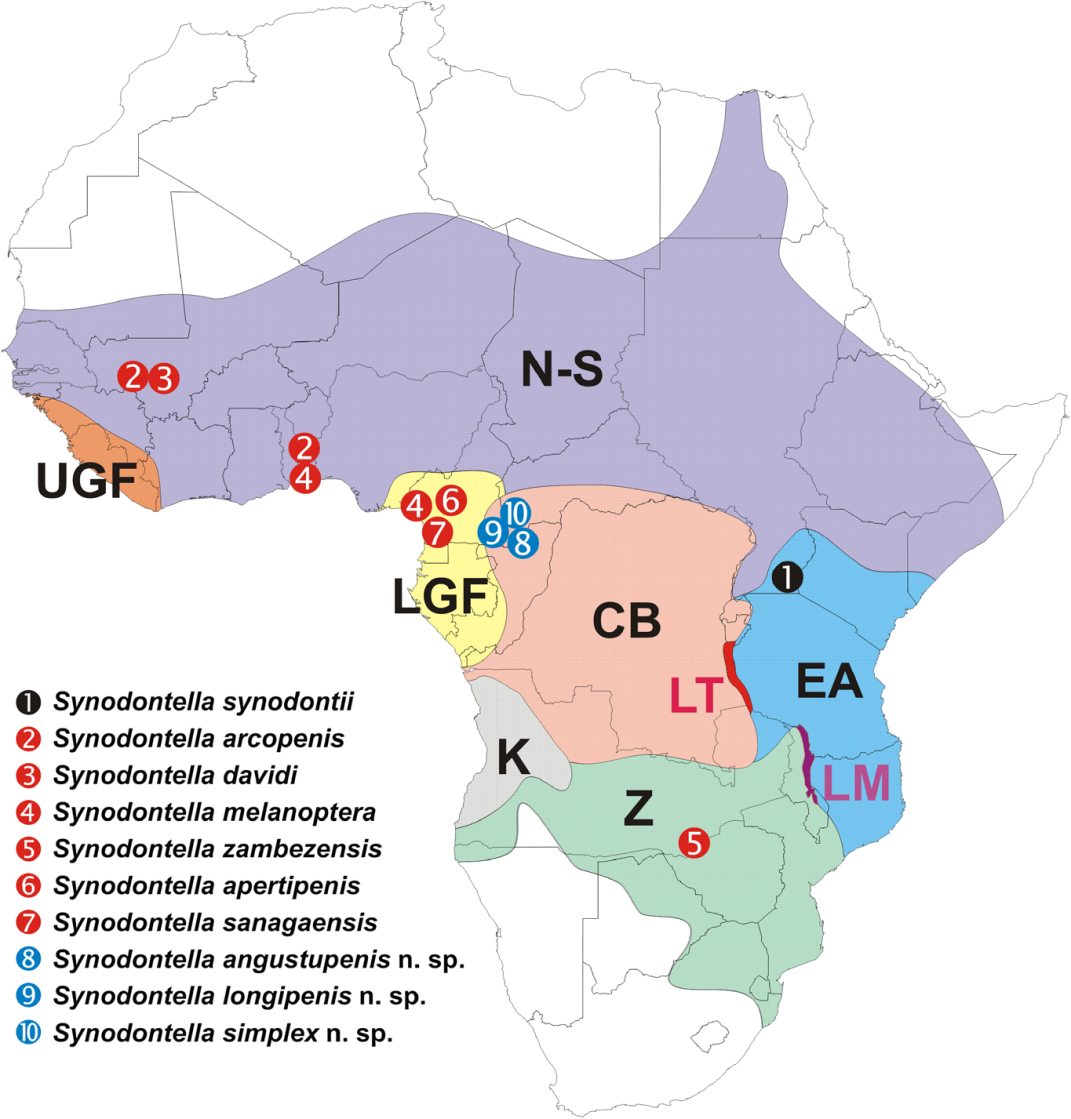
Map showing the major ichthyological provinces of Africa [Nilo-Sudan (N-S), Upper Guinea Forest (UGF), Lower Guinea Forest (LGF), Congo Basin (CB), Quanza (K), Zambezi (Z), East Africa (EA), Lake Tangayika (LT) and Lake Malawi (LM)], with the type localities of described *Synodontella* spp. (in blue: species with horseshoe-shaped dorsal bars) (modified after [14]).

Three new species are described in this study, resulting in a total of 10 species of *Synodontella* described on the gills of *Synodontis* fishes, all being oioxenous [6] toward their hosts. *Synodontella angustupenis* n. sp., *S. longipenis* n. sp. and *S. simplex* n. sp. appear to form a complex of morphologically related species characterized by having a horseshoe-shaped dorsal bar. To date, known Cameroonian *Synodontella* species can be divided in two subgroups (see Fig. 5). The first one includes *Synodontella* species with the dorsal bar slightly curved, parasites of *Synodontis* spp. from the Sanaga River (Low Guinea Forest Basin) (*S. apertipenis*, *S. sanagaensis* and *S. melanoptera*) [11]. The second subgroup is made up of the three new species of *Synodontella* described herein with a horseshoe-shaped dorsal bar, parasites of *Synodontis* spp. from Boumba River (Congo Basin). It is suggested that the difference between these monogenean species could result from the geographical isolation of the populations (hosts and parasites) from the Sanaga and Boumba systems. An ancestral host species through time could have fragmented into relatively large and isolated populations followed by lineage divergence of both parasite and host populations [2, 8] with the formation of allopatric descendant species. In this case, the present observation may serve as evidence for the existence of two or more genetic lineages within the genus *Synodontella*. A slightly curved dorsal bar is also present in *S. arcopenis* from the Ouémé (Benin), *S. davidi* from the Niger (Mali) and *S. zambezensis* (Zambia) (*S. synodontii* having a V-shaped dorsal bar). Therefore, the shape of the dorsal bar does not appear to be related to the geography of the hosts or parasites (Fig. 6), but the authors emphasize the potential importance of the dorsal bar in the classification of species of *Synodontella*, especially as host specificity imposes morphological adaptation of the attachment organs of parasites found on phylogenetically related hosts. In addition, the degree of host specificity is correlated with the morphological attributes of the parasite attachment organ [15], and thus may explain parasite diversification.
